# The prognosis and prevention measures for mental health in COVID-19 patients: through the experience of SARS

**DOI:** 10.1186/s13030-020-00196-6

**Published:** 2020-09-22

**Authors:** Guo Heng Mo, Zi Xuan Wang, Xiao Si Chen, Qunguang Jiang

**Affiliations:** 1 Department of Gastrointestinal Surgery, the First Affiliated Hospital of Nanchang University, Nanchang, 330006 Jiangxi People’s Republic of China; 2 Queen Mary College of Nanchang University, Nanchang, Jiangxi China; 3 The Fourth Clinical Medical College of Nanchang University, Nanchang, Jiangxi China; 4 The First Clinical Medical College of Nanchang University, Nanchang, Jiangxi China

**Keywords:** COVID-19, Mental health, Prognosis, Treatment, Depression

## Abstract

Due to the high pathogenicity and mortality, the COVID-19 disaster caused global panic and anxiety. At present, diagnosis and treatment are of great concern. As time progresses, however, the sequelae caused by many other organ system complications and treatments will become increasingly obvious, and psychosomatic symptoms are one of these changes with great potential impact. Studies have shown that symptoms like poor sleep quality, anxiety and even delirium are not uncommon in patients during isolation. By summarizing the follow-up study on mental and psychological health of SARS in the past 10 years, and combining the characteristics of the existing cases of COVID-19, we will provide suggestions for the prevention and treatment of psychological diseases in clinical practice.

## Background

Nearly 8 months have passed since the first confirmed case of Corona Virus Disease-2019 (COVID-19) was found in Wuhan, China, in December 2019, and now the world is threatened by this highly infectious and devastating viral pneumonia. As of 26th August 2020, around 23,965,059 people in the world have been infected with the virus, and this number is still surging every day in Europe and America, the case fatality rate even exceeded 5%. In China, the earliest outbreak point, although the infection has been basically controlled, a large number of discharged patients are facing the burden brought by the complications of various organ systems and the mental pressure from the change of social roles and environment. As for the 187 countries where the outbreak is still raging, effective mental health interventions are needed to respond to the treatment pain of a large number of isolated patients, the depression of severe patients and the psychological pressure of medical staff under overload work.

In view of the strong homology between the COVID-19 genome sequence and Severe Acute Respiratory Syndrome (SARS) [1], the currently reported cases of COVID-19 and SARS patients have great similarities in the symptoms of various organ systems such as pulmonary fibrosis, viral myocarditis, acute renal injury and other symptoms [2], and mental health is no exception [3]. In the follow-up study of SARS patients for nearly 10 years, it can be found that the mental symptoms will continue to exist after discharge and a considerable proportion of survivors will have mental diseases such as depression and Posttraumatic Stress Disorder (PSTD) in the recovery period due to the influence of social factors and identity changes [4]; moreover, severe patients, high-dose corticosteroids and the status of medical staff are independent predictors of mental illness. Since the transmissibility and duration of critically ill patients with COVID-19 are much higher than that of SARS [5, 6], it is reasonable to believe that under the long-term pressure of this epidemic on patients, medical staff and the health care system, there will be a larger population facing various potential pressures in the future, such as the persistence or even worsening of residual symptoms, complications and side effects of treatment, and the negative impact on their quality of life and social role function after returning to society.

Currently, we know little about the long-term impact of mental health of COVID-19 patients, so the importance of detection and treatment of comorbidity is self-evident, and mental health services can play an important role in rehabilitation. By summarizing the potential impact of major emergent events on mental health and behavioral symptoms such as anxiety, stress, fear, violence, progressive neurological dysfunction and cognitive impairment of survivors, and combining previous research and data characteristics of mental disorders caused by infectious diseases such as SARS, this paper concluded the mental and psychological symptoms that may appear on a large scale in the prognosis of patients with COVID-19 a highly dangerous disease spreading globally today, the risk factors and possible effective intervention programs. Then, it can further provide a comprehensive reference for the researchers in the field of global mental health and front-line workers, such as improving the medication plan in the treatment period, conducting certain psychological counseling as early as possible, and focusing on high-risk groups in the follow-up period of prognosis, moreover, regularly carrying out psychological assessment and psychological intervention treatment and giving certain drugs to control the further development of mental and psychological problems and the progressive damage of other organs for patients with severe emotional stress.

### The universality and severity of mental diseases

Excluding acute lung, heart, kidney and other organic damage, psychological damage may have a wide and long-term impact. The outbreak of SARS in 2003 can be regarded as a mental health disaster. PTSD is the most common chronic psychosis, followed by depression. The problem of mental illness after the epidemic is extremely serious, and its influence covers all fields and is closely related to everyone. In one follow-up study of 195 adult SARS patients, 10 to 18% reported PTSD 1 month after discharge, and the severity of symptoms was related to higher perceived life threat and lower emotional support [7]. Another study of 180 SARS survivors (average age 36.9 years) also pointed out that the psychological distress in them after 1 month’s recovery was real and significant, and negative assessment may play a key role in the development of psychological distress in these survivors. For instance, the negative appraisal of acute phase with significant influence was ‘passing the SARS virus on to the family’, whereas the convalescence after recovery was changed to ‘drug side-effects’ and ‘permanent damage to health’ [8]. For SARS patients, compared with a control group, the study showed that the stress level was higher during the outbreak and no sign of decline after 1 year.

SARS survivors also showed worrisome depression, anxiety and post-traumatic symptoms, with an alarming proportion (64%) of those who had reached the diagnostic criteria for psychosis [9]. More studies have shown that in the 4 years of SARS treatment, PTSD occurred in a high proportion (44.1%) of subjects who recovered from SARS, with persistent psychological distress and social function weakening [10], and such a huge psychological trauma was also observed in Middle East Respiratory Syndrome (MERS) [11]. However, mental and psychological stress and post-traumatic stress disorder not only cause great persistent damage to patients themselves, but also post serious threats to their contacts. Due to the pressure of contagion, people tend to vent their emotions to innocent people, and those who feel exposed to danger may have a strong tendency to insult others and even resort to violence against them [12], like what happened in Ukraine.

Notably, the mental health of survivors in terms of stress, anxiety, depression and post-traumatic symptoms did not improve over time, but gradually deteriorated [13]. The study found that most of the first symptoms of depression occurred in the late stage of treatment and 3 months after discharge, while the existence of some protective factors such as feeling lucky, increased civic awareness and a sense of solidarity, played a role in alleviating depression, but as the epidemic passed, the buffering effect gradually weakened, and then the depression continued to grow [8]. Another report also confirmed this: when life is no longer under imminent threat, other concerns gradually emerge, such as the fear in isolation ward, complications of SARS and their treatment (such as avascular necrosis of the femoral head and osteoporosis), discrimination, unemployment, economic pressure, or the threat of other subsequent outbreaks of infectious diseases that may or have occurred [14].

### The pathogenesis of mental diseases after highly infectious diseases

It is reported that neuroendocrine, neural structure and neuroimmune disorders play an important role in depression and PTSD. On the one hand, coronavirus can guide the immune damage mechanism in the body and induce a large number of inflammatory reactions. In the blood and Cerebrospinal fluid (CSF) of patients with coronavirus pneumonia, Interleukin-6(IL-6), C-reactive protein (CRP), Interleukin-1(IL-1), Tumor Necrosis Factor (TNF) and other proinflammatory factors are all increased to varying degrees [15], among with high levels of IL-6 are strongly related to progressive neurological dysfunction, neurodegeneration and cognitive impairment [16]. This probably the mechanism how the virus affects the patient’s mental health molecularly. And many evidences show that the inflammatory reaction in the lung, heart and nervous system will last for a long time in the prognosis of SARS [17], and lead to a variety of chronic inflammatory diseases such as pulmonary fibrosis, viral myocarditis, multiple sclerosis [18], while the chronic inflammation, especially the inflammatory complications of the nervous system related to the changes of brain structure and function, which is considered to be one of the pathogenesis of depression [19, 20]. On the other hand, the inflammatory reaction of the nervous system will cause the metabolic changes of the basal ganglia and affect the information processing through the cingulate cortex. Changes in the cingulate cortex may indicate increased sensitivity to conflict and negative life events [21], which further complements the possible pathogenesis of depression in patients with COVID-19 .

Secondly, in PSTD, the increase of corticotropin releasing factor (CRF) induced by traumatic stress may play an important role in the process of enhancing activated macrophage releasing facto [22], and these macrophages are widely distributed in the peripheral nervous system and brain, thus causing stress response to the decrease of stress threshold.

The long-term prognosis of COVID-19 patients with mental disorders after rehabilitation is similar to that of SARS and other major epidemics [23], and its severity and persistence are immeasurable. The study points out that with the delay of time and the deepening of symptoms, the proinflammatory cytokines in patients with depression, fatigue and severe insomnia will further increase, which will aggravate the complications of other organ systems [24, 25]. Long term depression is an important cause of cognitive decline and neurodegenerative change, and the activation of macrophages in the blood and microglia in the brain caused by chronic inflammation will further aggravate neurodegenerative change, which is closely related to the transformation from depression to dementia [19]. Therefore, extensive psychological treatment and counseling services need to be paid attention to and to strictly control the progress of mental and psychological diseases.

### High risk factors of mental disorders caused by COVID-19

In previous retrospective studies, the occurrence of psychosocial diseases is often strongly associated with specific population or special environmental factors. For example, although the stress level of medical staff among SARS survivors during the outbreak was similar to that of non-medical staff, a year later, the pressure level of medical staff was significantly higher, with higher levels of depression, anxiety, post-traumatic symptoms and scores. After controlling factors such as age, gender and education, health care workers are six times more likely to score above the General Health Questionnaire-12 (GHQ-12) threshold due to the dual pressure of being patients and medical staff [26], so the situation of SARS survivors among medical staff is particularly worrying and they need more attention [26]. 17.3% of the medical staff without the disease had similar mental symptoms [27], including chronic fatigue, pain, weakness, depression and sleep disorders [28]. Over the long term, of the 90 subjects recruited, a quarter had post-traumatic stress disorder (PTSD) 30 months after SARS, while 15.6% had depression [13]. What’s more, different occupations have different effects on mental health, doctors have more physical symptoms compared to nurses [29]. Our results, therefore, highlight the need to enhance the preparedness and capacity of healthcare professionals to detect and manage the psychological consequences of future comparable outbreaks of infectious diseases.

The infection of virus is not only the battle of patients and medical staff, but also the battle of the whole country and people, as it is closely related to everyone and no one can stay out of the business. Studies have showed that the residents in the areas with high prevalence of SARS, regardless of age, continued to develop more serious post-traumatic interference than those in the areas with low prevalence of SARS. In addition, the prevalence of PTSD was significantly higher in the elderly and residents in SARS epidemic areas [30]. The most relevant predictor is the financial stress caused by reduced income after illness, and other items included gender, range of activities, dietary restrictions, travel restrictions, clothing disinfection and infection control [31]. These different pressures are highly correlated with the psychological stress index, so the causes of psychological problems are not only isolated factors. During one-year follow-up period, evidence showed that both women and medical staff were risk factors for mental maladjustment. Female survivors had higher stress levels, more severe depression and anxiety, and more severe post-traumatic stress symptoms, and their GHQ-12 scores were three times higher than that of men. Among them, women and participants with low education level are more likely to have avoidance symptoms [7]. In a study of college students, the number of stressors and the use of avoidance strategies can positively predict psychological symptoms [32] . When controlling stressors, positive coping can positively predict life satisfaction [33]. Therefore, in the face of large-scale stressors such as COVID-19, increasing psychological counseling and treatment services will be of great significance.

We found that the high-risk factors for the prognosis of patients with COVID-19 are not only related to the specific population, but also highly related to the treatment drugs in the acute phase. For example, the use of a large number of interferon in the treatment of coronavirus will lead to emotional depression, anxiety, short-term memory disorders of sleep disorders through the impact on endocrine and changes in neurotransmitter and immune system changes [34]; meanwhile, high dose of exogenous steroids can cause reversible memory damage through the effect on hippocampal metabolism [35].

### The necessity of prevention of mental disorders in COVID-19 patients

From the current situation of transmission, we are concerned about the mental state of people around the world. With regard to SARS, whose outbreak was brought under control in a few months mainly due to the two characteristics of SARS coronavirus: First, coronavirus is a large virus and is not easy to mutate; second, the infected people show obvious symptoms when they are likely to spread the disease, so they can be identified and isolated in time [12]. However, for this new coronavirus disease, the mutation of the virus is currently unknown, but some studies believe that novel coronavirus in different regions has some genetic differences. Furthermore, Zhong Nanshan believes that the longest incubation period of coronavirus is 21 days, with an average incubation period of 7 days [36]. A large number of asymptomatic cases have been reported in many countries, which has brought great difficulties to identification and isolation while greatly increasing anxiety and fear in people. For these patients with severe acute respiratory syndrome, the dread of this new type of fatal infectious disease, the fear of relatives and friends’ infection, the experience of witnessing adverse events during hospitalization, the uncertainty of prognosis, and the nursing experience in Intensive care unit (ICU) are all terrible experiences. Under an overburdened medical system, followed by the contagion of stress in special occupational groups, especially for medical workers, inadequate personal protective equipment, unsystematic isolation process, and inactive disease control will make fear spread rapidly among individuals [12].

### Treatment suggestions for improving social psychological prognosis

Psychological intervention is very necessary for patients, health care workers and the general public. We should pay attention to the high-risk groups prone to disease, and at the same time eliminate the stimulation factors that induce PTSD and depression as much as possible, and timely and early intervention is beneficial to reduce the incidence of PTSD [37]. We should not be overly pessimistic the prognosis of patients with COVID-19, although part of the source of mental illness is poor prognosis [4]. For some drugs that cause long-term adverse physical conditions, such as hormones [35], they are abandoned or restricted in the treatment guidelines of COVID-19, and according to all materials known on PubMed, the newly add drugs have no clear long-term impact. According to statistics, effective antidepressants such as chronic antidepressants can greatly reduce the degree of depression and anxiety symptoms [38], its chronic effect is related to the repair and possible construction of injured neural network; moreover, it can promote the growth of axons and dendrites [39], and may also play a role in the organ injury caused by chronic inflammation. However, we are concerned about the psychological status of medical workers in some countries, because some studies have shown that during the outbreak, medical workers experienced anxiety, depression and poor sleep. Such situation began to improve 2 weeks after the adoption of SARS prevention and control measures, and systematic SARS prevention plan improved the mental state of medical workers [40]. At the same time, the country’s active and timely resource scheduling and integration is particularly important, and ecouraging the health care workers and improving the long-term potential for adverse mental health conditions is an important part of the country’s response to the epidemic [41].

It should be noted that stigmatization of disease may have a longer-term impact than disease itself. Two thousand three SARS virus, HIV and Spanish influenza, which was mistakenly associated with Spanish people, all of them have caused huge psychological and economic losses to specific groups and regions. The stigmatization of diseases, especially of certain ethnic groups, can lead to adverse social emotions and promote racism. In 2006, don C. des jarlais PhD proposed a positive and effective strategy for the United States to remove the stigma of Acquired Immune Deficiency Syndrome (AIDS) and protect people suffering from AIDS, such as the President of the United States posing with AIDS patients, and the promulgation of the Americans with Disabilities Act [42]. It is the common goal for all countries to pay attention to the prevention and treatment of diseases and take measures to protect the patients and it is also a task for every country to remove the stigma of disease. Therefore, in the COVID-19 treatment and prognosis stage, evaluation of psychological problems requires flexible skills due to subjects’ fear of stigmatisation.

Generally speaking, it is of great significance to carry out active and effective mental health education for the whole people, popularize relevant mental education assistance work as soon as possible, and provide timely psychological support with self-coping strategies to enhance their resilience and reduce their fear, anxiety and pressure. At the same time, it is more important to ensure the psychological status of specific occupations and high-risk groups, and the possibility of long-term subjective pain and occupational difficulties for medical workers infected with COVID-19 could be included in the data model, which will provide a better basis for future prevention and treatment [42]. Effective prior preparation, such as effective risk communication and the provision of psychological first aid before psychological stress and behavioral symptoms, can improve the effectiveness of post disaster intervention [12]. In addition, further longitudinal follow-up study on survivors can timely assess their mental consequences and determine the relationship between patients and their families’ mental health, and it is also an important way to prevent and early correct patients’ psychological and social stress during the follow-up period, and can provide a strong basis and reference for future emergent events.

## Conclusion

The mental and psychological impact of major public health events may be long-term and even far outweigh the initial threat to life. The psychological symptoms of COVID-19 patients and cured survivors include poor sleep quality, low mood, anxiety, inattention, and two of the most common and severe long-term mental illnesses-depression and PSTD, which will cause great burden and threat to society in the long run. Age, gender, incidence rate and risk factors of acute phase are highly correlated with psychological morbidity, thereby providing a reliable basis for early identification and psychological support. At the same time, medical staff, as a special group fighting against epidemics and susceptible to infection, are facing multiple and complex pressures and are more prone to mental and psychological diseases, especially in the infected medical staff. Inflammatory response and other organ system prognosis sequelae are the possible mechanism for the generation and continuous progression of depression and PSTD. Therefore, it will exert a significant effect by reducing the dosage of corticosteroids in the treatment period, actively treating the psychiatric or psychological complications in the rehabilitation period with psychological counseling and treating severe cases of depression with chronic antidepressant medication. In addition, the government should provide public policy support to survivors, de-stigmatize the disease, and develop follow-up research programs to strengthen the evaluation of COVID-19 for long-term mental illness.

## Data Availability

Not applicable.

## Abbreviations

COVID-19: Corona Virus Disease-2019
SARS: Severe Acute Respiratory Syndrome
MERS: Middle East Respiratory Syndrome
PSTD: Posttraumatic Stress Disorder
IL-6: Interleukin-6
CRP: C-reactive protein
IL-1: Interleukin-1
TNF: Tumor Necrosis Factor
CRF: Corticotropin-releasing factor
CSF: Cerebrospinal fluid
AIDS: Acquired Immune Deficiency Syndrome
ICU: Intensive care unit
GHQ-12: General Health Questionnaire-12
